# Decoding the role of extracellular vesicles in pathogenesis of cystic fibrosis

**DOI:** 10.1186/s40348-025-00190-4

**Published:** 2025-04-21

**Authors:** Priya Kalsi, Nikhil Gupta, Gitanjali Goyal, Himanshu Sharma

**Affiliations:** Department of Biochemistry, All India Institute of Medical Sciences, Bathinda, 151001 Punjab India

**Keywords:** Cystic fibrosis, Extracellular vesicles, miRNA, Oxidative stress

## Abstract

**Background:**

Intercellular communication is a critical process that ensures cooperation between distinct cell types and maintains homeostasis. In the past decades, extracellular vesicles (EVs) have been recognized as key components in cell-to-cell communication. These EVs carry multiple factors such as active enzymes, metabolites, nucleic acids and surface molecules that can alter the behavior of recipient cells. Thus, the role of EVs in exacerbating disease pathology by transporting inflammatory mediators, and other molecular signals that contribute to chronic inflammation and immune dysregulation in various diseases including cystic fibrosis (CF) is well documented.

**Main body:**

CF is a genetic disorder characterized by chronic inflammation and persistent infections, primarily affecting the respiratory system. This review explores the multifaceted roles of EVs in CF lung disease, focusing on their biogenesis, cargo, and contributions to disease progression. It is well known that CF results from mutations in the CFTR (cystic fibrosis transmembrane conductance regulator) gene, leading to defective ion transport, thick mucus secretion, and a propensity for bacterial infections. However, it has been observed that EVs derived from CF patients carry altered molecular cargo, including proteins, lipids, RNA, and DNA, which can exacerbate these conditions by promoting inflammation, and modulating immune responses. Beyond their pathogenic roles, EVs also hold significant therapeutic potential. Their natural ability to transfer bioactive molecules positions them as promising vectors for delivering therapeutic agents, such as gene therapy constructs and anti-inflammatory compounds. Accordingly, a study has shown that these EVs can act as a carrier molecule for transport of functional CFTR mRNA, helping to restore proper chloride ion channel function by correcting defective CFTR proteins in affected cells.

**Conclusion:**

This review aims to summarize the role of EVs and their molecular cargo in pathogenesis of CF lung disease via modulation of intracellular signaling leading to persistent inflammation and increased disease severity. We also explored the mechanisms of EV biogenesis, cargo selection, and their effects on recipient cells which may provide novel insights into CF pathogenesis and open new avenues for EV-based therapies aimed at improving disease management.

## Background

Cystic fibrosis (CF) is an autosomal recessive condition affecting multiple organs and tissues, characterized by persistent airway infections that ultimately lead to respiratory failure. It is a complex multisystem genetic disorder resulting from mutations in the Cystic fibrosis trans-membrane conductance regulator (CFTR) gene located on chromosome 7q31.2. This gene encodes the CFTR protein, an ATP-binding cassette (ABC) transporter that plays a critical role in ion transport across epithelial membranes. The activity of the CFTR protein is regulated by cAMP-mediated phosphorylation via protein kinase A (PKA) and intracellular ATP, ensuring proper ion and fluid homeostasis [1, 2]. CFTR is an anion channel mainly conducting Cl^−^ ions across the epithelial cell apical membrane of lungs, upper respiratory tract, pancreas, liver, gallbladder, intestines, sweat glands, and reproductive tract [1]. It also regulates the transmembrane flow of other electrolytes such as HCO3−, Na+, and K + ions, which help to control the viscosity of mucus in the airway epithelial cells [3, 4].

According to the current statistics from Cystic Fibrosis Mutation Database [5], more than 2000 CFTR gene variants have been reported, with Phe508del as most common mutation which affect majority of CF population [3, 4, 6]. Most of the mutations in the CFTR gene results in non-functional CFTR ion channel, and affects fluid transport through the airway epithelium, resulting in the accumulation of thick, viscous mucus. The increased viscosity of airway mucus leads to the obstruction of small and medium-sized bronchioles and bronchiectasis, leading to bacterial infection and inflammation, which can further progress to respiratory failure and death [4, 6].

Extracellular vesicles (EVs) are small sized membrane bound vesicles released from all cell types and play a crucial role in intercellular communication. EVs have been found to significantly mediate intercellular information transfer through multiple pathways, including direct contact and signal molecule transmission. EVs also facilitate the transport of various molecules, leading to altered cellular metabolism including activation of inflammatory response and cellular proliferation [7]. In accordance, one of the studies, have reported that the exosomes (a type of EVs) derived from human and murine B lymphocytes can induce antigen-specific major histocompatibility complex (MHC) class II restricted T cell responses and participate in antigen presentation in vivo [8, 9]. In recent years, the involvement of EVs in various pathological conditions has been elucidated, revealing that their specific cargo is linked to different pathophysiological states [10, 11]. For example, in CF airways, neutrophil infiltration is a characteristic feature and it was observed that neutrophils are highly activated when exposed to EVs isolated from CFTR-mutant cells. Also, these EVs were found to promote neutrophil migration, increase their size and granularity, and elevate markers such as RAGE, ERK, and p38 in stimulated neutrophils [11]. In addition, studies have documented the role of EVs and its cargo in inflammatory responses, and production of pro inflammatory cytokines with both beneficial and detrimental effects in other organs such as heart, and liver [12, 13].

Hence, given the integral role of EVs in the pathogenesis of various diseases, researchers have increasingly focused on understanding the involvement of EVs in the progression of CF lung disease [1, 14, 15]. The exosomes released from CF epithelial cells contain different protein content as compared to those released by healthy cells. Various studies have investigated EVs in bodily fluids of CF patients, highlighting their role in disease pathogenesis [1, 14, 15]. A proteomic study on CF bronchoalveolar lavage fluid (BALF) revealed elevated levels of grancalcin, histones, and inflammation-related proteins like LCN2 and S100A12, along with antioxidant defense proteins such as SOD2 and GPX3 [15, 17]. Further, In-vitro studies have also shown that EVs released from CFTR-deficient airway cells were significantly higher in number and contributed to excessive neutrophil activation via RAGE-S100A12 interaction [6]. In addition, CF sputum-derived EVs trigger inflammasome activation in epithelial cells, and leads to inflammation in the CF lungs [6, 16, 17]. In view of the diversified role on EVs, this review highlights the crucial involvement of EVs in pathogenesis and progression of CF lung disease, focusing on role of EVs in induction of inflammation, oxidative stress and activation of EV-miRNA mediated signaling pathways leading to influence CF pathophysiology. Further we have also explored the upcoming therapeutic potential of EVs in rescuing CFTR associated defects.

## Pathogenesis of CF lung disease

CF is a multisystem disorder primarily caused by the mutations in the CFTR gene. The disease manifests only when an individual inherits two pathogenic variants of the CFTR gene, one from each parent [18]. Based on their impact on the level of protein function, pathogenic CFTR gene variants are categorized into seven classes (I to VII) [19]. It is notable that F508del, which is the most common lethal CFTR gene mutation belongs to the class II category. These class II CFTR variants are associated with altered structural conformation, and this misfolded CFTR proteins is degraded by the chaperons mediated Endoplasmic Reticulum-Associated Degradation (ERAD) pathway. As a result, the mutant CFTR protein fails to reach the cell surface, leading to a ‘trafficking defect' [1, 20]. Furthermore, the functionally impaired CFTR protein leads to ameliorated mucociliary clearance, creating an environment suitable to chronic bacterial colonization. Subsequently, it results in persistent infections which trigger an exaggerated inflammatory response, characterized by neutrophilic infiltration and the release of pro-inflammatory cytokines, further damaging the airway epithelium [1, 2]. In few studies, it is also suggested that defective CFTR reduces anion chloride ion (Cl−) secretion while increasing ENaC (epithelial sodium channel)-mediated Na⁺ absorption in the airway, contributing to airway surface dehydration [3, 18, 21]. The non-functional CFTR also disrupts the normal conductance of bicarbonate (HCO₃⁻) ions and indirectly modulates HCO₃⁻/Cl⁻ exchangers, leading to an abnormally low airway lumen pH. As a result of this micro-environment imbalance the activity of antimicrobial peptides is impaired, leading to elevated inflammatory response and susceptibility to bacterial infections [22]. Moreover, chronic bacterial infections also play a major role in the pathogenesis of CF lung disease, exacerbating lung damage and accelerating disease progression. For instance, *Pseudomonas aeruginosa* is the most common pathogen, often leading to persistent lung infections that promote inflammation, biofilm formation, and airway obstruction, which ultimately result in lung function decline [23, 24]. Hence, these interconnected processes such as bacterial infections, mucus obstruction, inflammation, and structural airway damage highlight the multifactorial nature of CF pathogenesis, resulting in the progressive decline in pulmonary function [25].

### EVs in pathogenesis of CF lung disease

EVs have emerged as pivotal mediators in the pathogenesis of CF lung disease, contributing to the intricate interplay among chronic inflammation, infection, and cellular dysfunction [4, 11]. These nanoscale particles, carry a diverse array of bioactive cargo, that influence intercellular communication and modulate key processes in CF lung disease progression. Hence, by facilitating cross-talk between CFTR deficient epithelial cells and other neighbouring cells, EVs play a critical role in perpetuating the pathological cycles of CF [26].

**Fig. 1:**
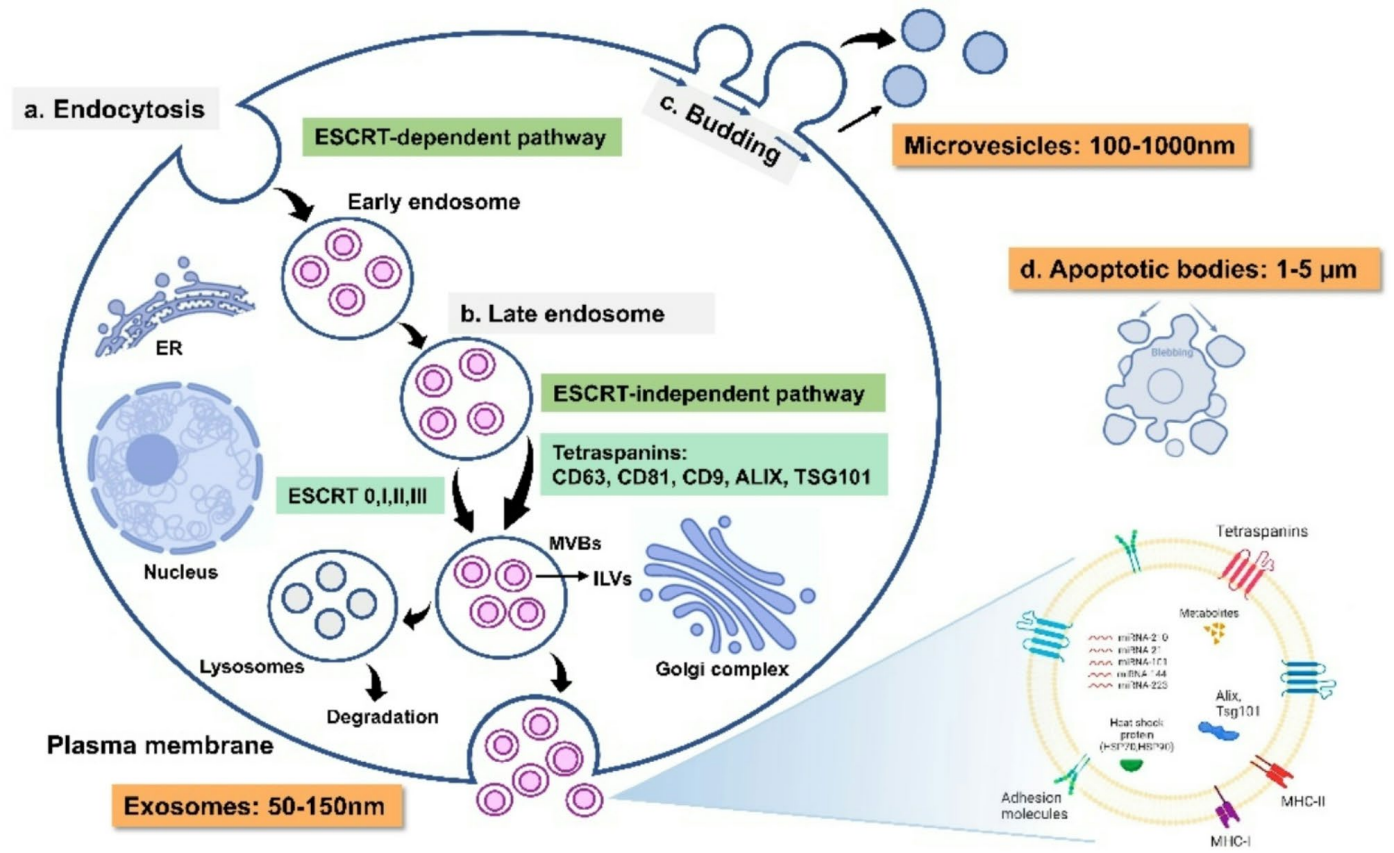
Biogenesis of EVs **a** The formation of EVs starts with endocytosis, leading to formation of early endosomes and late endosomes **b** Late endosomes then convert into MVBs through either ESCRT-dependent or ESCRT-independent pathways **c** MVs (100–1000 nm) form by budding directly from the plasma membrane **d** Apoptotic bodies (1–5 μm) are produced during cell apoptosis via blebbing

EVs are a heterogenous family of membrane vesicles and contains various proteins, lipids and nucleic acids. EVs can be categorized into the exosomes (50–150 nm), microvesicles (MVs, 100–1000 nm), and apoptotic bodies (1–5 μm), with sizes ranging from 50 nm to 10 μm [27]. Each of these types are different and has its own functions and characteristics. For instance, the exosomes are recognized for their involvement in intercellular communication, biological signal transduction, regulation of cellular behavior, and immunomodulatory responses [28]. Whereas, the apoptotic bodies are known to be involved in the horizontal transfer of oncogenes, DNA, and the presentation of T cell epitopes and immunosuppression when they were taken up by phagocytes [9, 29]. All these EVs are produced through distinct pathways depending on their sub-type, for instance, the exosomes originate from early endosomes, which form through plasma membrane internalization. Within these endosomes, molecules are sorted into intraluminal vesicles (ILVs) in multivesicular bodies (MVBs), which fuse with the plasma membrane to release the exosomes. This process is regulated by ESCRT-dependent and non-ESCRT-dependent mechanisms involving tetraspanins [30]. Whereas microvesicles are formed by outward budding of the plasma membrane, driven by cytoskeletal rearrangements mediated by actin, myosin, and calcium signaling. Also, ESCRT-III and lipid redistribution play key roles in their release [31]. Further, apoptotic bodies are generated during cell apoptosis through membrane blebbing, packaging apoptotic DNA, RNAs, and cytoplasmic components, facilitating the clearance of apoptotic cells by phagocytes. Each type of EVs carries specific cargo influenced by its biogenesis and parent cell, contributing to intercellular communication in physiological and pathological processes (See Fig. 1.).

In recent years, the role of EVs in the pathogenesis and progression of various diseases including CF has been extensively studied. Studies have investigated the presence of EVs in bodily fluids of CF patients and discovered their significant role in the disease’s pathogenesis [4, 32]. For instance, a proteomic study on bronchoalveolar lavage fluid (BALF) from CF patients revealed that the exosomes isolated from respiratory tract from these patients had elevated levels of grancalcin and histones, crucial for neutrophil adhesion, degranulation, and antimicrobial activity [15]. Also, it was observed that these EVs contained higher levels of inflammation-related proteins, such as neutrophil gelatinase-associated lipocalin (LCN2) and S100A12 and were rich in antioxidant defense proteins like superoxide dismutase (SOD2), glutathione peroxidase 3 (GPX3), and peroxiredoxin 5 (PRDX5) [15, 33]. The probable role of EVs in CF lung disease pathogenesis was also reported in an in-vitro study by *Useckaite et al.*,* 2020*, which has shown that the EVs released from CF airway cell lines were significantly higher in number compared to wild-type controls [6]. These EVs were taken up by healthy donor neutrophils, increasing their CD66 expression, and myeloperoxidase activity [6]. More recently, *Forrest* and colleagues in 2021 demonstrated that EVs isolated from sputum of CF patients can activate naïve neutrophils, inducing exocytosis of primary granules and production of caspase-1 and IL-1β [16]. Activated neutrophil-derived EVs deliver active caspase-1 to primary tracheal epithelial cells, activating the inflammasome and releasing IL-1α, IL-1β, and IL-18 [16]. Also, there have been studies which show that EVs from neutrophils can cause activation of other neutrophils, and can down-regulate their ability to kill bacterial pathogens [16, 34]. Due to the high abundance of EVs in sputum, inflammasome, these neutrophil-derived EVs have emerged as critical mediators of pathologic signaling in CF and other neutrophilic airway diseases [16]. Further, in a study by *Katja et al.*,* 2023*, It was highlighted that EVs from airway epithelial cells (AEC) play a crucial role in modulating immune responses by monocyte-derived macrophages (MDM) [35]. In CF, EV-mediated signaling is impaired due to mutations in CFTR, leading to attenuated cytokine secretion and reduced immune cell recruitment. This defective cross-talk between AEC and MDM exacerbates immune dysfunction in CF, contributing to the pathogenesis of the disease, particularly in response to bacterial infections like *Pseudomonas aeruginosa* [35].

### Bacterial EVs and CF lung disease

CF is associated with a reduced volume of airway surface liquid overlaying pulmonary epithelial cells, increased accumulation of viscous mucus, and the entrapment of pathogens. Furthermore, Common pathogens in CF lungs include *Pseudomonas aeruginosa (P. aeruginosa)*,* Staphylococcus aureus (S. aureus)*,* and Haemophilus influenzae (H. influenzae)*. Among these, *P. aeruginosa* colonizes approximately 50% of adults as well as paediatric CF patients [36, 37]. Despite intensive antimicrobial therapy, *P. aeruginosa* persists, and leads to increased exacerbations, progressive decline in lung function, and thus contributes as a leading cause of high morbidity and mortality in individuals with CF [38, 39]. Notably, *P. aeruginosa* primarily resides in the mucus layer overlaying lung epithelial cells and secretes EVs that diffuse through this mucus barrier. These EVs fuse with airway cells, delivers virulence factors and other toxins into the host cell cytoplasm, thereby modifies the innate immune response in CF patients [40, 41]. For instance, gram-negative bacterial derived EVs, such as those secreted by *P. aeruginosa*, contain lipopolysaccharide (LPS), a known virulence factor on their surface, contributing to the pathophysiology of the disease [40, 42]. Moreover, in a study by *Hendricks et al.*,* 2021*, EV mediated transfer of essential nutrients from host airway epithelial cells to *P. aeruginosa* was observed, hence EVs also serve as crucial nutritional factor for propagation of *P. aeruginosa*, and pathogenesis of CF [43]. Furthermore, the increased levels of miR-223-3p or miR-451a, along with decreased levels of miR-27b-3p in sputum supernatants, were also found to be correlated with pulmonary exacerbations in children with CF infected by Aspergillus, Haemophilus, and Pseudomonas, respectively [43]. Hence, the interplay between bacterial cell secreted EVs, and those released from CFTR-deficient cells in CF patients, emphasize their significance in progression of CF lung severity. Therefore, EVs play a multifaceted role in the pathogenesis of CF lung disease, acting as key mediators in the chronic inflammation, as well as infection. Their diverse cargo and biogenesis pathways contribute to pathological signalling, particularly in neutrophilic inflammation and immune dysfunction. Literature have highlighted the involvement of EVs in promoting neutrophil activation, modulating immune responses, and facilitating microbial interactions, further perpetuating the cycles of inflammation and infection characteristic of CF lung disease [4, 16].

## Inflammatory response in CF lung disease

The inflammatory response is a complex process and involves multiple mechanisms to defend against pathogens and tissue repair. In the lungs, it has been observed that inflammation is usually caused by the pathogens or by exposure to the toxins, pollutants, and allergens. During inflammation, various types of inflammatory cells become activated, and each releasing cytokines and mediators that influences the activities of other inflammatory cells. Clinically, acute inflammation is seen in conditions such as pneumonia and acute respiratory distress syndrome (ARDS), whereas chronic inflammation is hallmark of CF, asthma and chronic obstructive pulmonary disease (COPD) [44, 45]. The inflammatory response in CF airways occurs early in the disease process. Increasing evidence suggests that CFTR dysfunction itself drives a dysregulated inflammatory response and that, before any infection, CF airways are already in a proinflammatory state that provides a substrate for subsequent tissue damage and chronic infection [46, 47]. When pathogens colonize the dysfunctional airway microenvironment, activation of host protective mechanisms, including release of proteases, reactive oxygen/nitrogen species (ROS/RNS), and proinflammatory chemokines by epithelial and inflammatory cells, is exaggerated, and may cause tissue damage [48, 49]. Beyond the role of cytokines and immune cells in CF-associated inflammation, emerging evidence also highlights the contribution of dysregulated circulating miRNAs as key regulators of this process [50–52]. These miRNAs influence the expression of inflammatory mediators and pathways, contributing to the chronic inflammatory environment of CF lung disease [50–52]. Studies highlighted the regulatory influence of dysregulated miRNAs on the expression of the CFTR mRNA, as well as their involvement in modulating inflammation within CF airways [53, 54]. A variety of dysregulated miRNAs have been shown to modulate the expression of key pro-inflammatory mediators such as IL-6 and IL-8. For instance, high levels of miR-146a and miR-199a-5p exacerbate inflammation by promoting overproduction of pro-inflammatory cytokines such as IL-6 and upregulating Toll-like receptor 4 (TLR4) expression. Conversely, reduced levels of miR-17 and miR-93 are associated with increased IL-8 production, a key driver of neutrophil recruitment in CF airways. These findings emphasize the critical contribution of miRNA dysregulation to the chronic inflammatory environment characteristic of CF [54–59].

### EV cargo mediated inflammatory response in CF lung disease

Moreover, studies have shown that apart from common causes, EVs also play a crucial role in modulation of CF associated chronic inflammation [4, 60, 61]. Among the diverse cargo of EVs, miRNAs have been emerged as key modulator of cellular pathways in recipient cells. These EV derived miRNAs may activate inflammatory pathways and alter immune response in recipient cells and thus play a key role in disease pathogenesis [62]. In a study by *Stachowiak et al.*,* 2023*, EV-derived miRNAs isolated from serum and sputum of CF paediatric CF patients were found as mediators of pulmonary exacerbation and were associated with inflammation and tissue remodeling [63].

In addition to miRNAs, the protein cargo of EVs also plays a significant role in the pathogenesis of CF lung disease. Research has shown that the exosomes derived from CF lung epithelial cells exhibit a distinct protein composition compared to EVs released by healthy cells [64]. EVs contribute to inflammation in CF by modulating the migration and activation of neutrophilic leukocytes within the airways. *Useckaite* and colleagues in year 2020, examined the content of EVs from CF bronchial epithelial cells (CFBE41o-) and found that these vesicles were notably enriched with proteins linked to acute inflammatory stress and infection in CF lung disease [6]. Also, enrichment of proteins such as VCAM1, with consequent migration of neutrophils to inflammatory sites, was also observed in EVs isolated from CF airway cell lines [6]. In addition, pathway analysis of proteins cargo in EVs isolated from BALF of CF patients revealed activation of cytokine signaling in the CF airways. Further, calgranulins such as S100A, S100A9 and S100A12 (another family of pro-inflammatory proteins) were also found to be elevated in EVs isolated from sputum of CF patients. Evidences indicate that these proteins can activate bronchial epithelial cells through TLR4, elicit ERK phosphorylation, facilitate NF-κB translocation to the nucleus and induce MUC5AC production, all of which are key features of CF lung inflammation [65, 66]. More studies further confirmed the presence of increased levels of S100A12 in CF patients which results in the migration of leukocytes to the inflammation sites [6, 67]. Similarly, another study by *Vazquez et al.*,* 2023*, identified high levels of proteins in EVs from CF patients that were involved in the activation of NF-κB pathway suggesting that EVs are involved in the proinflammatory propagation in CF [67]. While research on role of EV mediated cargo is still an ongoing process, some evidences suggest that EVs under MV category which are derived from granulocytes of CF patients have also been associated with an extensive presence of neutrophils in the CF airways [68–71]. Simultaneously in another study it has been suggested that higher levels of PD-L1 in EVs from fibroblasts upon TGF-β stimulation could contribute to immune suppression and fibrogenesis [70, 72]. Hence, the evidence underscores the significant role of EV cargo in the pathogenesis of CF airways through various mechanisms. The analysis of EV cargo offers potential for targeted therapeutic interventions, providing valuable insights into the inflammatory response involved in CF lung disease.

## EV mediated induction of oxidative stress in CF airways

Oxidative stress is defined as an imbalance between increased reactive species (ROS and RNS) and reduced or defective antioxidant mechanisms. This imbalance leads to oxidative damage, which is known to increase with age and is associated with various diseases [73]. Reactive oxygen species (ROS) and reactive nitrogen species (RNS), which are oxygen and nitrogen derivatives respectively, are generated through aerobic metabolism and play crucial roles in both normal physiological functions and pathological processes. While ROS and RNS are essential for physiological processes such as cell signaling and immune responses, their excessive levels can damage cellular components like DNA, proteins, and lipids [73]. However, to counteract the high ROS levels, the body uses various antioxidant mechanisms, including enzymes such as superoxide dismutase (SOD), catalase, and glutathione peroxidase (GPX). Despite the potential oxidative damage from excess ROS levels, it has been studied that they also play essential roles in normal physiological functions. For instance, low levels of H_2_O_2_ are crucial for cellular processes like proliferation, differentiation, migration, and apoptosis. However, excessive ROS can damage DNA and contribute to tumor development and progression by modifying signaling pathways, such as MAPK/ERK, PI3K/AKT, and NF-κB [74, 75].

Given the well-documented role of oxidative stress in the pathogenesis of various diseases, its contribution to the progression of lung injury in CF patients is also well-established in the literature. Studies have shown that CF patients experience a significant deficit in antioxidant molecules and subsequently an increase in oxidative stress [76, 79]. This sustained imbalance between oxidants and antioxidants induces chronic inflammation, a key factor contributing to persistent cellular damage and obstructing proper airway remodeling [76]. Further, studies have shown that, in CF patients, malabsorption of dietary antioxidants due to exocrine pancreatic insufficiency and the inability of CFTR-mutant cells to efflux glutathione (GSH) also play a crucial role in exacerbating systemic redox imbalance [77, 78]. Therefore, chronic oxidative stress has become a primary determinant of progressive lung damage in CF, leading to pro-inflammatory environment [78, 79].

Furthermore, studies have shown that the EV cargo in CF patients is altered, with EV-associated miRNAs differing from those in non-CF individuals [6, 15]. Since, EV mediated miRNAs have been increasingly recognized for their role in the progression of various pathological conditions by mediating pathways such as NF-κB, TGF-β, and Notch signaling, highlighting their role in oxidative stress, disease progression, and immune activation [80, 81], we hypothesize that altered EV-associated miRNAs may similarly influence redox pathways in CF, potentially exacerbating oxidative damage and inflammation. However, no such studies have been reported and further research is required to confirm this hypothesis and determine whether EV-mediated miRNAs contribute to oxidative stress and CF lung pathology.

**Fig. 2:**
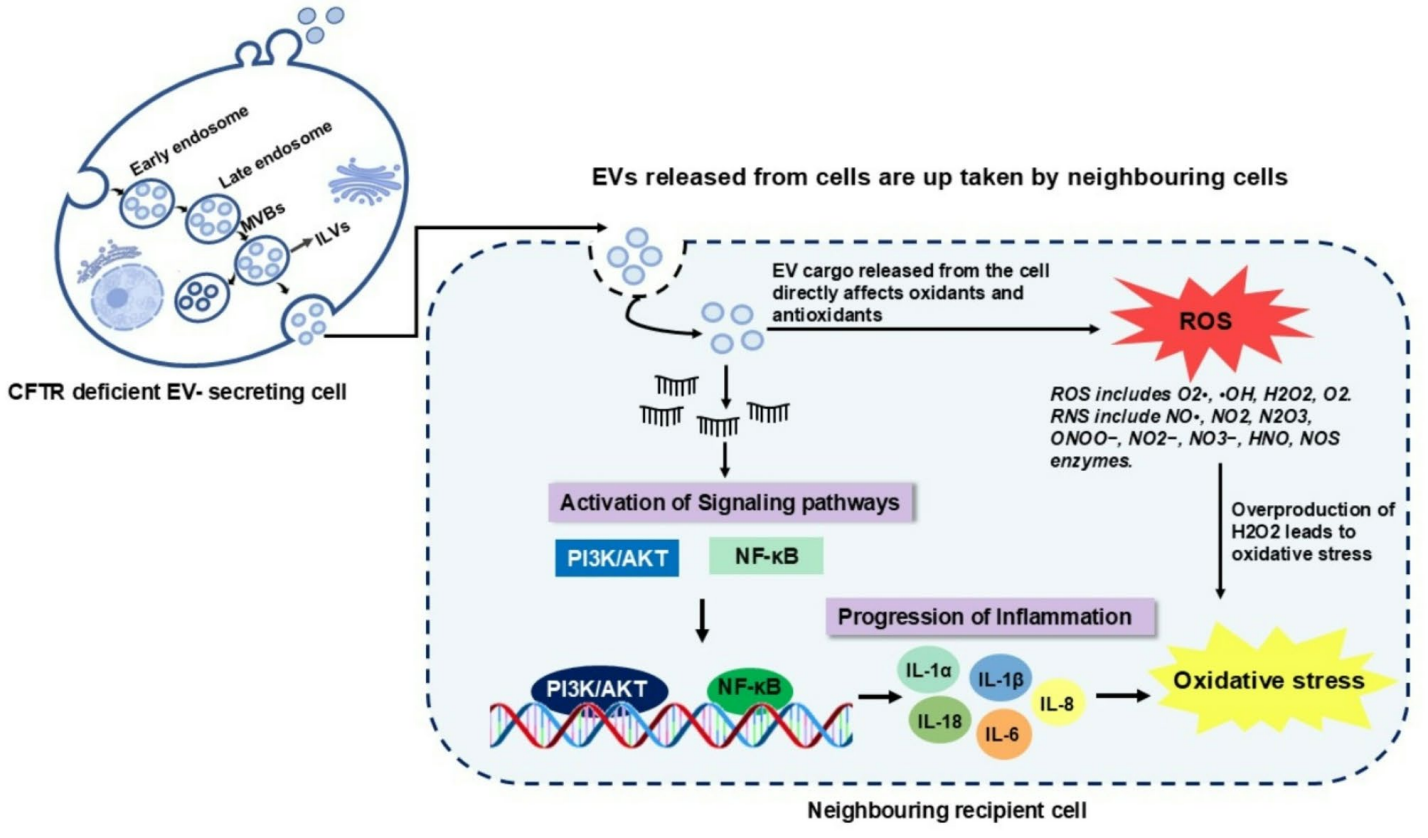
EV mediated alteration in molecular pathway leading to increased oxidative stress in recipient cell. CFTR-deficient cells secrete altered EVs and trigger inflammation in the neighboring recipient cells by activation of the inflammatory signaling pathways and causing oxidative stress through increased production of reactive oxygen species (ROS) and reactive nitrogen species (RNS)

In view of this, in one of the studies, it was found that EVs collected from respiratory tract of CF patients were rich in SOD2 and GPX3, two proteins contributing to antioxidant defense [15]. These proteins were found to be upregulated under pro-inflammatory conditions. However, SOD2 is highly expressed in CF epithelia, leading to the overproduction of H_2_O_2_, which triggers the activation of the NF-kappa B pathway (See Fig. 2). Further, this deleterious effect is balanced by the peroxidase activity of another molecule GPX3, which catalyzes the reduction of organic hydroperoxides and hydrogen peroxide by glutathione, protecting cells against oxidative damage [15, 82]. Thus, oxidative stress is considered a key player in the progression of various diseases, including CF. The chronic imbalance between oxidant and antioxidant molecules in CF leads to persistent inflammation and lung damage. While the role of EVs in mediating oxidative stress and contributing to the proinflammatory environment in CF has been suggested, research specifically exploring this connection remains limited. Therefore, understanding the interplay between oxidative stress and EVs in CF lung pathogenesis need further research and may present a promising avenue for developing targeted therapies to alleviate the disease burden.

## Therapeutic potential of EVs

**Fig. 3:**
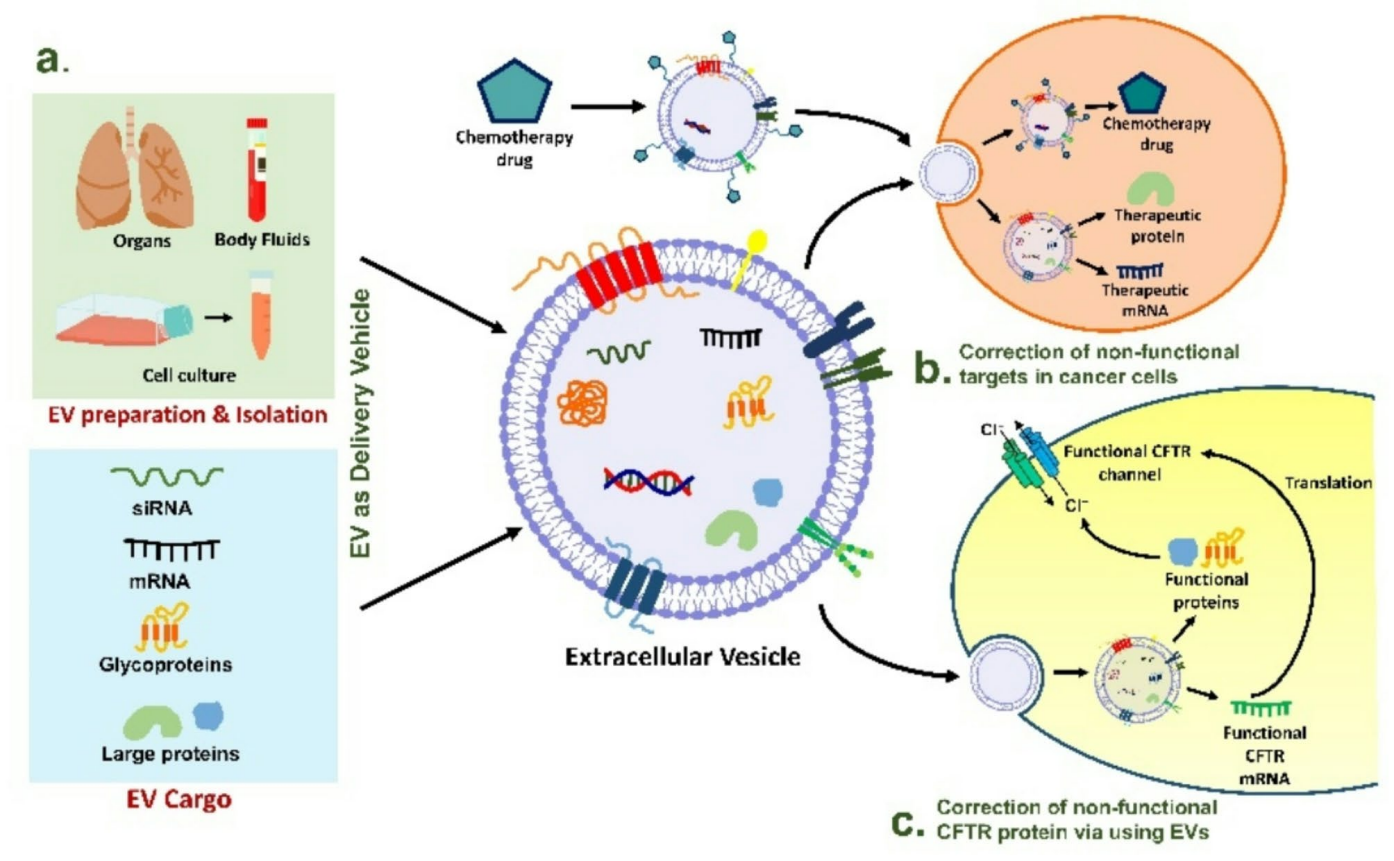
Role of EVs as therapeutic molecule **a** EVs can be isolated from various sources and these vesicles carry diverse molecular cargo, including siRNA, mRNA, large functional proteins. These vesicles serve as natural delivery vehicles, capable of transporting therapeutic agents such as chemotherapeutic drugs, mRNA, or proteins to target cells. **b** EVs as carrier molecule for delivery of therapeutic agents to diseased cells **c** EVs as carrier molecule for transport of functional CFTR mRNA, helping to restore proper chloride ion channel function by correcting defective CFTR proteins in affected cells

Besides the role in the pathogenesis of various diseases, EVs have gained significant attention for their therapeutic implications [83]. Recent investigations have highlighted the significance of EVs in the treatment of various pathological conditions. Due to their ability to transfer bioactive molecules and cross biological barriers, EVs are now extensively studied by researchers as potential therapeutic tools [83, 84]. Hence, various studies have discovered the potential use of EVs as therapeutic agents not only in diseases like cancer, neurological disorders, cardiovascular and lung diseases but also in rare disorders like CF [85, 86]. In accordance, *Gonzalez* and colleagues in 2012 proved the hypothesis by successfully delivering functional human CFTR glycoprotein and CFTR mRNA using the exosomes as a delivery agent in Chinese hamster ovarian cells [87]. To explore this further, *Vituret* and colleagues in 2016 investigated the use of MVs and exosomes as carriers to deliver exogenous CFTR glycoprotein and its encoding mRNA to human CF cells, aiming to restore CFTR chloride channel function [34]. In their study, MVs and exosomes were isolated from the culture medium of CFTR-positive Calu-3 cells, which had been transduced with an adenoviral vector overexpressing GFP-tagged CFTR (GFP-CFTR). Both MVs and exosomes had the capacity to package and deliver the GFP-CFTR glycoprotein and mRNA-GFP-CFTR to target cells in a dose dependent manner [34, 88]. Additionally, in another study it was demonstrated the use of EVs to manipulate gene expression in an *in- vitro* airway model. It was reported that MV-mediated delivery of CFTR protein was significant in correction of the transepithelial Cl^−^ current in well-differentiated HAE from CF donors [88]. Further, investigators also tested the use of EVs for delivering CFTR-modulating agents, such as zinc finger protein activators to enhance gene expression [89]. In a study by *Villamizar et al.*,* 2021*, stromal-derived mesenchymal stem cells (MSCs) were engineered to produce EVs containing CFTR zinc finger (CFZF) protein fused with transcriptional activation domains, facilitating CFTR promoter activation and transcription in human bronchial epithelial cells (HuBEC) [90]. These engineered EVs successfully delivered CFZF-VPR to HuBEC, restoring CFTR expression. Further, *Zulueta* and colleagues in 2018 treated IB3-1 CF cell lines with EVs derived from human lung MSCs and observed that the administration of MSC derived EVs resulted in downregulation of the transcription and protein expression of pro-inflammatory cytokines IL-1β, IL-8, and IL-6 [91]. Also, there was an upregulation of PPARγ mRNA expression, a transcription factor that controls anti-inflammatory and antioxidant mechanisms through NF-κB and HO-1 pathways. In addition, there was a significant reduction in NF-κB nuclear translocation alongside an increase in HO-1 expression, indicating a clear disruption of the downstream inflammatory cascade [91, 92]. Hence, based on these limited finding, EVs demonstrate significant therapeutic potential not only by acting as a carrier for delivery of genetic material but also by managing chronic inflammatory response in CF. Traditional gene delivery methods, such as viral vectors (adenovirus, lentivirus, and AAV), have been widely used; however, these are often limited by time-consuming production, high costs, and potential immunogenicity. In contrast, EVs offer several advantages, including enhanced biocompatibility, serum stability, and reduced clearance by major organs. Notably, the exosomes have been shown to effectively deliver small molecules such as siRNAs and drugs, while larger microvesicles (MVs) have demonstrated the ability to transfer functional CFTR protein to CF airway cells. Given these benefits, EV-based delivery systems represent a promising avenue for CF therapeutics, with potential applications in gene modulation, targeted drug delivery, and correction of CFTR defects [34] (See Fig. 3).

## Conclusion

**Fig. 4:**
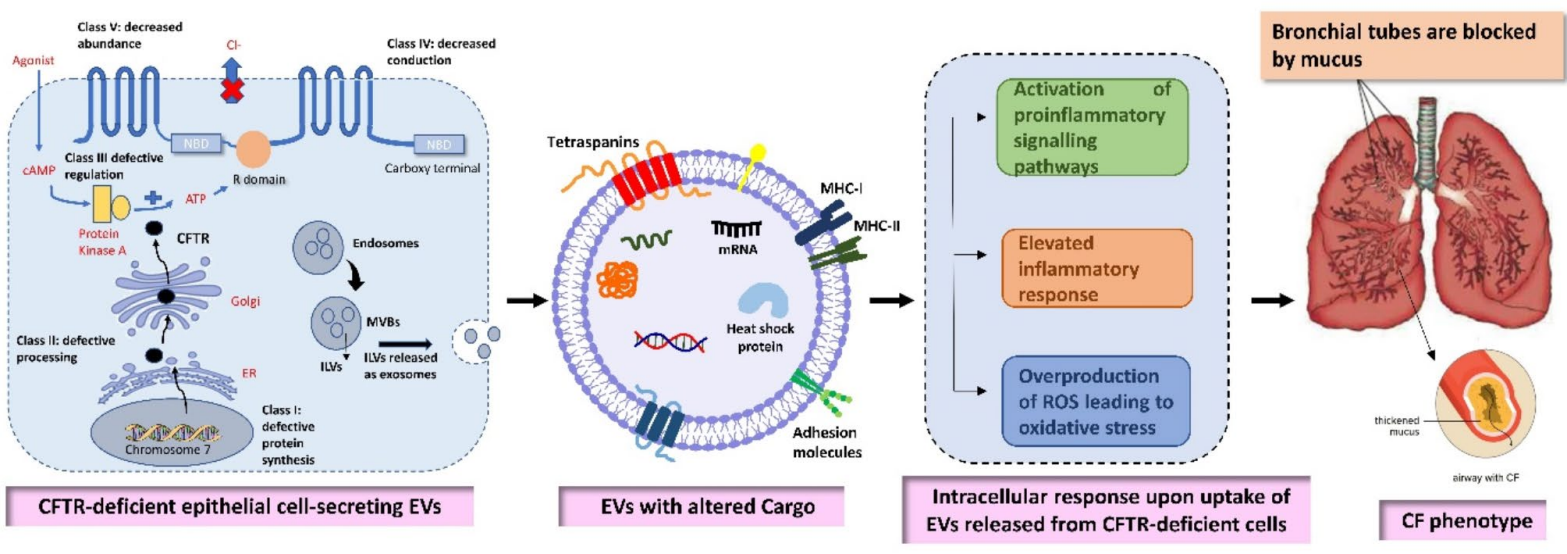
Role of EVs in pathogenesis CF. CFTR deficient cells release EVs consisting with altered molecular cargo leading to activation of proinflammatory signaling pathways and inducing oxidative stress in the recipient pulmonary cells, and hence exacerbated pulmonary function leading to CF phenotype

Emerging research consistently highlights the critical role of EVs in the progression of various diseases such as CF. EVs, key players in intercellular communication, carry a variety of bioactive molecules, including proteins, lipids, and nucleic acids. Various studies have confirmed the relation between EV cargo and severity of the CF. These vesicles have ability to influence the behavior of recipient cells through altered mechanisms, autocrine and paracrine signaling, contributing to the complex inflammatory landscape in CF. Increasing evidences suggest that several mechanisms have been identified by which EVs exacerbate inflammation, hence leading in the severity of the disease. For instance, studies have showed that the cargo of EVs have potential to provoke pulmonary exacerbation and leads to progression of CF lung disease. Furthermore, it is evident that EVs can induce oxidative stress by delivering reactive oxygen species or by modulating the antioxidant defenses of recipient cells. This oxidative stress further exacerbates cellular damage and inflammation. Signaling pathways activated by EVs also play a crucial role in provoking inflammation in CF patients. The receptor for advanced glycation end products (RAGE) and the nuclear factor kappa B (NF-κB) pathways are particularly noteworthy in the patients having CF. EVs can activate RAGE, leading to the subsequent activation of NF-κB, a pivotal transcription factor that drives the expression of different pro-inflammatory cytokines and chemokines. This cascade significantly contributes to the chronic inflammatory state observed in CF lungs. Given the critical role of EVs and their cargo in the CF pathogenesis, these vesicles hold promise as a potential therapeutic target (See Fig. 4). However, despite the advances in understanding the role of EVs in CF, significant gaps remain in our knowledge, particularly regarding the mechanistic interplay between different cell types and the specific cargo of EVs in the progression of CF as well as various other pathological conditions. Hence, addressing these unresolved challenges is essential for enhancing our understanding and translating it into clinical applications that can substantially improve patient outcomes. Future research that delves into these interactions and thoroughly characterizes EV cargo may reveal new therapeutic targets to reduce the inflammatory burden in CF and enhance patient care. Therefore, it is essential to understand and investigate the intricate mechanism of EVs and its cargo.

## Data Availability

No datasets were generated or analysed during the current study.

## Abbreviations

EVs: Extracellular Vesicles
CF: Cystic Fibrosis
CFTR: Cystic Fibrosis Transmembrane Conductance Regulator
DNA: Deoxyribonucleic Acid
RNA: Ribonucleic Acid
MHC Class II: Major Histocompatibility Complex Class II
ESCRT: Endosomal Sorting Complex Required for Transport
BALF: Bronchoalveolar Lavage Fluid
LCN2: Lipocalin 2
SOD2: Superoxide Dismutase 2
GPX: Glutathione Peroxidase
PRDX5: Peroxiredoxin 5
RAGE: Receptor for Advanced Glycation End products
IL: Interleukin
ABC Transporter: ATP-Binding Cassette Transporter
ERAD: Endoplasmic Reticulum-Associated Degradation
ENaC: Epithelial Sodium Channel
CFRD: Cystic Fibrosis-Related Diabetes
PWCF: People with Cystic Fibrosis
MVs: Microvesicles
MVBs: Multivesicular Bodies
ILVs: Intraluminal Vesicles
ARDS: Acute Respiratory Distress Syndrome
COPD: Chronic Obstructive Pulmonary Disease
TNF-alpha: Tumor Necrosis Factor Alpha
CXCL: C-X-C Motif Chemokine Ligand
LPS: Lipopolysaccharide
TLR4: Toll-Like Receptor 4
VCAM-1: Vascular Cell Adhesion Molecule 1
NF-κB: Nuclear factor kappa-light-chain-enhancer of activated B cells
MUC5AC: Mucin 5AC
TGF-beta: Transforming Growth Factor Beta
ROS: Reactive Oxygen Species
RNS: Reactive Nitrogen Species
SOD: Superoxide Dismutase
GSH: Glutathione
PRDX: Peroxiredoxin
MSCs: Mesenchymal Stem Cells
GFP: Green Fluorescent Protein
HAE: Human Airway Epithelial
PE: Prolyl endopeptidase
AAV: Adeno associated virus
HuBEC: Human bronchial epithelial cells
CFZF- VPR: Cystic Fibrosis Zinc Finger-VP64-p65-Rta
HO-1: (Heme Oxygenase-1)
